# The prevalence and risk factors of school absenteeism due to premenstrual disorders in Japanese high school students—a school-based cross-sectional study

**DOI:** 10.1186/s13030-016-0067-3

**Published:** 2016-04-26

**Authors:** Mari Tadakawa, Takashi Takeda, Yasutake Monma, Shoko Koga, Nobuo Yaegashi

**Affiliations:** Department of Obstetrics and Gynecology, Tohoku University Graduate School of Medicine, 1-1 Seiryocho, Aobaku, Sendai, Japan; Division of Women’s Health, Research Institute of Traditional Asian Medicine, Kindai University School of Medicine, 377-2 Ohno-Higasi, Osaka-Sayama, Osaka 589-8511 Japan; Graduate Medical Education Center, Tohoku University Hospital, 1-1 Seiryocho, Aobaku, Sendai, Japan; Gynecology Clinic Koga, 2-5-12 Kashiwagi, Aobaku, Sendai, Japan

**Keywords:** Premenstrual syndrome, Premenstrual dysphoric disorder, Japanese high school students, Adolescence, School absenteeism, Salty food, Exercise

## Abstract

**Background:**

Premenstrual disorders such as premenstrual syndrome (PMS) and premenstrual dysphoric disorder (PMDD) interfere with the daily lives of adolescents. The causes of PMS and PMDD are unknown, but lifestyle habits, such as regular exercise and taste preference are known to be associated. This study was conducted to investigate how premenstrual symptoms affect the school life in Japanese high school students and whether there was a risk factor for school absenteeism that is dependent on the types of premenstrual symptoms or lifestyle habits.

**Methods:**

A school-based survey was conducted in Sendai, an industrial city in Japan. A total of 901 girls aged 15–19 with regular menstrual cycles were assessed using the self-reporting premenstrual symptoms questionnaire (PSQ) and questions regarding school absence, taste preference, and exercise. We classified the girls into ‘no/mild PMS’, ‘moderate-to-severe PMS’ and ‘PMDD’ according to the PSQ. The girls were classified into the ‘absent’ group if they were absent for more than 1 day per month. We used multivariate logistic analysis to examine the risk factors for school absenteeism.

**Results:**

The rates of ‘moderate-to-severe PMS’ and ‘PMDD’ were 9.9 and 3.1 %, respectively. A total of 107 girls (11.9 %) were classified into the ‘absent’ group. Significant differences were observed in the prevalence of all premenstrual symptoms (*p* < 0.001), ‘age’ (*p* < 0.001), ‘a preference for salty food’ (*p* = 0.001), and ‘lack of regular exercise’ (*p* = 0.03) between the ‘absent’ and ‘non-absent’ groups. Multivariate analysis revealed that premenstrual symptoms such as ‘insomnia or hypersomnia’ (odds ratio [OR] 2.27, 95 % confidence interval [CI]: 1.46–4.17) and ‘physical symptoms’ (OR 2.24, 95 % CI: 1.37–3.66), ‘reduced social life activities’ (OR 2.71, 95 % CI 1.31–5.59), and ‘a preference for salty food’ (OR 1.89, 95 % CI: 1.20–2.98) were risk factors for school absenteeism.

**Conclusions:**

One in nine Japanese female high school students were absent from school due to premenstrual symptoms. Physical premenstrual symptoms and lifestyles, such as a preference for salty food and a lack of regular exercise, were identified as risk factors for school absenteeism.

## Background

Premenstrual syndrome (PMS) is characterized by emotional, behavioral, and physical symptoms that occur during the late luteal phase of the menstrual cycle and are relieved after the onset of menstruation. Epidemiological studies have reported that 5–8 % of reproductive age women exhibit moderate-to-severe premenstrual symptoms that interfere with their daily activities [1]. Other studies have suggested that more than 20 % of fertile women have premenstrual symptoms that cannot be clinically ignored [2]. A severe form of PMS has been classified as premenstrual dysphoric disorder (PMDD) according to the Diagnostic and Statistical Manual of Mental Disorders, fifth edition (DSM-5) [3]. Such premenstrual disorders were originally considered to begin in the early twenties in women [4]. However, recent studies have demonstrated that they start earlier in the teenage years [5, 6].

Adolescence is a unique time in human development that produces significant psychological and physiological changes. Dysmenorrhea is a known health concern in adolescence. In our previous study, we reported a correlation between dysmenorrhea and PMS/PMDD in Japanese high school students [7]. We also showed that the frequencies of ‘moderate-to-severe PMS’ and ‘PMDD’ in Japanese high school students were 11.6 and 2.6 % higher, respectively, than that of adult women (5.3 and 1.2 %) [5]. These findings suggested that PMS and PMDD are major problems that affect the daily lives of adolescents, possibly to a larger extent than adults. Although studies have been conducted to investigate the prevalence of PMS and PMDD in adolescents [8–11], no study has ever revealed how premenstrual symptoms actually affect the school or social life of girls in adolescence.

The causes of PMS and PMDD have not yet been clearly elucidated, but they have been suggested to include hormonal changes, neurotransmitters, stress, and lifestyle habits [1, 4]. Lifestyle habits involve factors such as diet and exercise. The relationship between PMS and diet, for example, calcium intake [8, 12], caffeine-containing beverages [13, 14], and chocolate or sweets containing refined sugar [15], has been reported. Many studies demonstrated that exercise could be used as a treatment for premenstrual disorders [16, 17], and these findings indicated an association between PMS and lack of exercise.

The aim of this study was to investigate how premenstrual symptoms affect the school and social life of Japanese high school students, with a focus on its influence on school attendance. We also compared the types of premenstrual symptoms and lifestyle habits of ‘absent’ girls and ‘non-absent’ girls to determine the risk factors for school absenteeism.

## Methods

This study was conducted in accordance with the principles outlined in the Declaration of Helsinki. Our Institutional Review Board at Tohoku University approved the study.

### Participants

A school-based survey was conducted in October 2009 using a sample of Japanese female high school students who belonged to four public high schools in Sendai, an industrial city in Japan. We recruited students, aged 15–19, with regular menstrual cycles (22–35 days) who were able to provide informed consent.

We have already reported the preliminary data obtained from one of these schools [5].

### Questionnaire

We used the Premenstrual Symptoms Questionnaire (PSQ), which was developed in our previous study [18], to screen for premenstrual symptoms. The PSQ translates the DSM-4 criteria [19] into a rating scale with degrees of severity described in Japanese and is essentially identical to the Premenstrual Symptoms Screening Tool [20]. The PSQ asked, “Within the last three months, have you experienced the following premenstrual symptoms starting during the week before menses and remitting a few days after the onset of menses?” The premenstrual symptoms listed in the PSQ were ‘depressed mood’, ‘anxiety or tension’, ‘tearful’, ‘anger or irritability’, ‘decreased interest in work, home, or social activities’, ‘difficulty concentrating’, ‘fatigue or lack of energy’, ‘overeating or food cravings’, ‘insomnia or hypersomnia’, ‘feeling overwhelmed’, and ‘physical symptoms such as tender breasts, feeling bloating, headache, joint or muscle pain, and weight gain’. The PSQ also asked whether such premenstrual symptoms interfered with ‘work efficiency or productivity, home responsibility’, ‘social life activities’, or ‘relationships with coworkers or family’. The PSQ asked participants to rate the degree of severity of symptoms into four grades: ‘not at all’, ‘mild’, ‘moderate’, and ‘severe’. We classified participants into three groups: ‘PMDD’, ‘moderate-to-severe PMS’, and ‘no/mild PMS’ based on the criteria suggested by Steiner et al. [20], as used in our previous study. [18]. We defined the ‘PMDD’ girls as those who reported at least one of the four core symptoms (‘depressed mood’, ‘anxiety or tension’, ‘tearful’, and ‘anger or irritability’) as severe and at least four additional symptoms (for a total of five) as moderate to severe. They also had to report that their symptoms interfered severely with their ability to function in at least one of three domains (‘work efficiency or productivity, home responsibility’, ‘social life activities’, ‘relationship with coworkers or family’). The ‘moderate-to-severe PMS’ group, who marginally missed fulfilling the DSM criteria for the diagnosis of PMDD, were girls who reported at least one of the four core symptoms as moderate to severe and at least four additional symptoms as moderate to severe. They also reported that their symptoms interfered moderately or severely with their ability to function in at least one of the three domains listed above. In addition to the PSQ, we asked the participants whether they were absent from school due to the previously described premenstrual symptoms and the number of days per month they were absent in the last three months. We classified participants into the ‘absent’ group if they had more than 1 day of absence per month and the others into the ‘non-absent’ group, as defined in previous studies [21].

We also asked participants the following questions in order to establish their lifestyle habits, such as taste preferences and the extent of daily exercise: ‘Do you regularly exercise (more than once per week)?’, ‘Do you prefer sweets?’, ‘Do you prefer salty food?’, and ‘Do you drink coffee?’. The questions were answered as either ‘yes’ or ‘no’. The questionnaire consisted of 19 questions.

### Statistical analysis

Data were analyzed statistically using the Statistical Analysis System 9.1 edition for WINDOWS (SAS Institute Inc, Cary, NC). We also used multivariate logistic analysis to examine the risk factors for school absenteeism. *P* < 0.05 was adopted as the level of significance.

## Results

A total of 1909 female students answered the questionnaire, with 1008 being excluded from the analysis because of incomplete data. Therefore, we analyzed the data of 901 girls aged 15–19 years. The background characteristics of the participants are described in Table 1. Eighty-nine participants (9.9 %) were classified as having ‘moderate-to-severe PMS’ and 28 (3.1 %) as having ‘PMDD’. The prevalence of each premenstrual symptom is shown in Table 2. More than half of the participants had ‘anxiety or tension’ (66.7 %), ‘anger or irritability’ (64.0 %), ‘difficulty concentrating’ (59.5 %), ‘fatigue or lack of energy’ (70.9 %), ‘overeating or food cravings’ (52.8 %), and ‘physical symptoms’ (60.9 %). Premenstrual symptoms impaired ‘work efficiency or productivity, home responsibility’ in 50.7 % of the participants, ‘social life activities’ in 23.3 %, and ‘relationships with coworkers or family’ in 24.0 %.

**Table 1:** Background characteristics of the participants (*N* = 901)

Characteristics	
Age, years ± SD	17.0 ± 1.0
Regular exercise habits, no. (%)	490 (54.4)
Prefer sweets, no. (%)	712 (79.0)
Prefer salty food, no. (%)	351 (39.0)
Drink coffee, no. (%)	662 (73.2)

*SD* standard deviation

**Table 2:** Prevalence rates of premenstrual symptoms and induced interference with work, usual activities, or relationships (*N* = 901)

Symptoms	Not at all	Mild	Moderate	Severe
Premenstrual symptoms, no. (%)				
Depressed mood	459 (50.9)	262 (29.1)	136 (15.1)	44 (4.9)
Anxiety or tension	300 (33.3)	324 (36.0)	196 (21.8)	81 (9.0)
Tearful	490 (54.4)	217 (24.1)	130 (14.4)	64 (7.1)
Anger or irritability	324 (36.0)	304 (33.7)	196 (21.8)	77 (8.5)
Decreased interest in work, home, or social activities	608 (67.5)	198 (22.0)	77 (8.5)	18 (2.0)
Difficulty concentrating	365 (40.5)	370 (41.1)	129 (14.3)	37 (4.1)
Fatigue or lack of energy	262 (29.1)	340 (37.7)	226 (25.1)	73 (8.1)
Overeating or food cravings	425 (47.2)	243 (27.0)	155 (17.2)	78 (8.7)
Insomnia or hypersomnia	461 (51.2)	243 (27.0)	133 (14.8)	64 (7.1)
Feeling overwhelmed	622 (69.0)	195 (21.6)	63 (7.0)	21 (2.3)
Physical symptoms	352 (39.1)	283 (31.4)	182 (20.2)	84 (9.3)
Interference with work, usual activities, or relationships, no. (%)				
Work efficiency or productivity, home responsibility	445 (49.3)	316 (35.1)	110 (12.2)	30 (3.3)
Social life activities	691 (76.7)	148 (16.4)	44 (4.9)	18 (2.0)
Relationships with coworkers or family	685 (76.0)	165 (18.3)	41 (4.6)	10 (1.1)

A total of 107 girls (11.9 %) were classified into the ‘absent’ group, which demonstrated that premenstrual symptoms affected school attendance. Table 3 shows the number of days of absence: 94 participants were absent for 1–3 days, 8 participants for 4–6 days, and 5 participants for 7–10 days. No participants were absent for more than 10 days. The mean number of absent days for all participants was 0.3 days per month, with 0.2 days for ‘no/mild PMS’, 0.9 days for ‘moderate-to-severe PMS’, and 0.9 days for ‘PMDD’. Although the prevalence of absent girls increased based on the severity of PMS, 64 (8.2 %) participants with ‘no/mild PMS’ were classified into the ‘absent’ group, which revealed that premenstrual symptoms are associated with school absenteeism not only in over-moderate PMS and PMDD groups, but also in participants with ‘no/mild PMS’.

**Table 3:** Days of absence per month due to premenstrual symptoms

Absent	Total	No/mild PMS	Moderate-to-severe PMS	PMDD
	*N* = 901	*n* = 784	*n* = 89	*n* = 28
Yes	107 (11.9)	64 (8.2)	31 (34.8)	12 (42.9)
1–3 day(s)	94 (10.4)	58 (7.4)	26 (29.2)	10 (35.7)
4–6 days	8 (0.9)	5 (0.6)	1 (1.1)	2 (7.1)
7–10 days	5 (0.6)	1 (0.1)	4 (4.5)	0 (0.0)

Significant differences were observed in the prevalence of all premenstrual symptoms (*p* < 0.001) between the ‘absent’ group and the ‘non-absent’ group. Among background and lifestyle risk factors, ‘age’ (*p* < 0.001), ‘regular exercise habits’ (*p* = 0.03) and ‘a preference for salty food’ (*p* = 0.001) were also significantly different (Table 4). Multivariate logistic analysis revealed that ‘a preference for salty food’ (odds ratio [OR] 1.89, 95 % confidence interval [CI]: 1.20–2.98) and premenstrual symptoms such as ‘insomnia or hypersomnia’ (OR 2.27, 95 % CI: 1.32–3.38), ‘physical symptoms, such as tender breasts, feeling bloating, headache, joint or muscle pain, and weight gain’ (OR 2.24, 95 % CI: 1.35–3.67) and ‘reduced social life activities’ (OR 2.71, 95 % CI: 2.71–5.59) increased the risk of school absence (Table 5).

**Table 4:** Comparison between the ‘absent’ group and ‘non-absent’ group

	Absent group *n* = 107	Non-absent group *n* = 794	*P*-value
Background and lifestyle risk factors, no. (%)			
Age, years ± SD	17.0 ± 1.0	16.6 ± 1.0	<0.001*
Regular exercise habits	47 (43.9)	443 (55.8)	0.03*
Prefer sweets	85 (79.4)	627 (79.0)	1.000
Prefer salty food	58 (54.2)	293 (36.9)	0.001*
Drink coffee	36 (33.6)	204 (25.7)	0.09
Premenstrual symptoms (over moderate severity), no. (%)			
Depressed mood	47 (43.9)	133 (16.8)	<0.001*
Anxiety or tension	62 (57.9)	215 (27.1)	<0.001*
Tearful	53 (49.5)	141 (17.8)	<0.001*
Anger or irritability	64 (59.8)	209 (26.3)	<0.001*
Decreased interest in work, home, or social activities	32 (29.9)	63 (7.9)	<0.001*
Difficulty concentrating	41 (38.3)	125 (15.7)	<0.001*
Fatigue or lack of energy	71 (66.4)	228 (28.7)	<0.001*
Overeating or food cravings	48 (44.9)	185 (23.3)	<0.001*
Insomnia or hypersomnia	57 (53.3)	140 (17.6)	<0.001*
Feeling overwhelmed	27 (25.2)	57 (7.2)	<0.001*
Physical symptoms	64 (59.8)	202 (25.4)	<0.001*
Induced interference with work, usual activities, or relationships (over moderate severity), no. (%)			
Work efficiency or productivity, home responsibility	41 (38.3)	99 (12.5)	<0.001*
Social life activities	28 (26.2)	34 (4.3)	<0.001*
Relationships with coworkers or family	23 (21.5)	28 (3.5)	<0.001*

*Statistically significant (*p* value < 0.05)

*SD* standard deviation

**Table 5:** Multivariate analysis of risk factors for school absenteeism due to premenstrual symptoms

Risk factors	OR	95 % CI
Background and lifestyle risk factors		
Age (+1 age)	1.34	1.01–1.79
Regular exercise habits	0.73	0.46–1.16
Prefer sweets	1.04	0.60–1.83
Prefer salty food	1.89^a^	1.20–2.98
Drink coffee	1.14	0.70–1.85
Premenstrual symptoms (over moderate severity)		
Depressed mood	1.37	0.73–2.53
Anxiety or tension	0.99	0.53–1.85
Tearful	1.28	0.68–2.42
Anger or irritability	1.58	0.86–2.90
Decreased interest in work, home, or social activities	1.83	0.99–3.40
Difficulty concentrating	1.12	0.63–1.97
Fatigue or lack of energy	1.61	0.91–2.85
Overeating or food cravings	0.85	0.5–1.43
Insomnia or hypersomnia	2.27^a^	1.46–4.17
Feeling overwhelmed	0.79	0.40–1.55
Physical symptoms	2.24^a^	1.37–3.66
Induced interference with work, usual activities, or relationships (over moderate severity)		
Work efficiency or productivity, home responsibility	1.82	0.85–3.90
Social life activities	2.71^a^	1.31–5.59
Relationships with coworkers or family	1.26	0.69–2.29

*OR* odds ratio, *95 % CI* 95 % confidence interval, ^a^statistically significant

## Discussion

Apart from our previous study [5], epidemiological studies have revealed a high prevalence of PMS and PMDD in adolescents: Steiner et al. [9] reported the prevalences as 8.3 % for PMDD and 21.3 % for severe PMS, whereas Drosdzol et al. [11] reported them as 4.17 % for PMDD and 76.39 % for PMS. In the present study, the prevalences of ‘PMDD’ and ‘moderate-to-severe PMS’ were 3.1 and 9.9 %, respectively. Moreover, Delara et al. [22] reported a significantly poor health-related quality of life in Iranian girls with PMDD using an index score from the Short Form Health Survey. However, to the best of our knowledge, no study has established the actual effects of premenstrual disorders on the school or social lives of adolescents.

Here, we present the first school-based survey to show how premenstrual disorders affect the school and daily lives of adolescents. According to the results obtained in this study, 11.9 % of Japanese high school students were absent from school due to premenstrual symptoms and up to 5.2 % of girls were absent for 2 days or more per month. This result supports the recent findings [9, 11, 22] of studies in which premenstrual disorders were shown to be major problems in adolescence that markedly interfered with school and social activities. Significant differences were observed in the prevalence of all premenstrual symptoms between the ‘absent’ group and the ‘non-absent’ group in the present study. ‘Absent’ girls consisted not only of participants that were classified with ‘moderate-to-severe PMS’ or ‘PMDD’, but also of a high rate of participants with ‘no/mild PMS’. This result indicated that premenstrual symptoms may lead to school absenteeism in any girl that has menses and ovulates. An analysis of factors interfering with work, activities, and relationships showed that ‘reduced social life activities’ was a risk factor for school absenteeism, conversely indicating that girls in the ‘absent’ group were having problems not only in their school lives but also in other social activities, such as hobbies or after-school activities.

A limitation of our study was that this was a retrospective, self-reporting design. Therefore, recall bias should be considered. Because menstruation-related symptoms differ to some extent in each cycle, some participants may have also had difficulties in accurately answering the questions. This may explain the high frequency of incomplete data noted in the present study. The low percentage of response (901/1909) should also be considered as a limitation. However, the rate of PMS severity was similar to that in our previous studies (prevalence of ‘moderate-to-severe PMS’ and ‘PMDD’ were 11.8 and 2.6 % in 2010 [5], and 11.3 and 3.2 % in 2012 [7], respectively). Therefore, we think that the data in the present study are reliable. To exclude bias due to educational levels, we recruited participants from four high schools of various levels. However, we should include high schools from other areas to examine the true prevalence of premenstrual symptoms and its effect on Japanese adolescent students.

The causes of PMS and PMDD have not yet been clarified but are believed to include lifestyle factors, such as diet and exercise. Previous studies have suggested associations between PMS and caffeine intake [13, 14], chocolate consumption [15], and exercise [16, 17]. However, few of these have been evaluated systematically. In our study, no correlations were observed between school absence and ‘a preference for sweets’ (*p* = 1.00) or ‘drinking coffee’ (*p* = 0.09), but a correlation was observed for ‘regular exercise habits’ (*p* = 0.03) and ‘a preference for salty food’ (*p* = 0.001). The association with the habit of regular exercise supports the findings of previous studies [16, 17]. Exercise may improve premenstrual symptoms mainly in emotional and physical conditions by increasing beta-endorphin levels and physical well-being [22]. Previous studies have reported the effectiveness of exercise towards physical symptoms, including breast tenderness and fluid retention symptoms [17]. In contrast, although no study has yet demonstrated that ‘a preference for salty food’ is associated with premenstrual disorders, decreasing salt intake in the diet has been one of the dietary treatments of premenstrual disorders [23]. In this study, a correlation was observed between ‘a preference for salty food’ and school absenteeism. We suggest that body water is retained when salty food is eaten, which may lead to physical symptoms such as headaches, weight gain, or edema. We previously demonstrated that physical premenstrual symptoms were related to the high frequency of school absenteeism, and this may be the reason for the higher OR of ‘a preference for salty food’ than those of other risk factors in lifestyle habits. On the other hand, a preference for salty food may be an aspect of western eating habits, including the daily consumption of junk food and snacks. Eating habits have become more western over the past several decades in Japan, and younger generations consume larger amounts of salty snacks and junk food than older generations [24]. The results from this study may increase awareness that eating habits and increasing consumption of salty snacks or junk food may have a prominent impact on the gynecological health of Japanese women, nevertheless, further investigation is required.

Medications such as serotonin selective reuptake inhibitors (SSRIs) have been the first choice to treat severe PMS and PMDD in adults. However, data on adolescents is lacking. Because SSRIs are difficult to prescribe to adolescents, other approaches towards stress, diet, and exercise may be important. Stress and a poor understanding of the dynamic changes occurring in adolescence may play a role in the development of premenstrual disorders. Therefore, education on the effects of ovarian hormones and menstrual cycles on emotional and physical aspects may be helpful to reduce stress related to menstruation, increase awareness, and increase the predictability of menstruation-related problems [6]. Chau et al. reported a significant improvement in knowledge and PMS scores after an educational program for teenage girls [25]. Moreover, guiding school girls with exercise and proper eating habits, including the reduced consumption of salty snacks or junk food, may lead to an improvement in physical premenstrual symptoms, which consequently may improve the school and social life qualities of adolescents.

## Conclusions

In total, 11.9 % of Japanese female high school students were absent from school for more than 1 day per month due to premenstrual symptoms. Premenstrual symptoms, such as ‘insomnia or hypersomnia’ and ‘physical symptoms such as tender breasts, feeling bloating, headache, joint or muscle pain, and weight gain’ were risk factors for school absenteeism. In addition, ‘a preference for salty food’ and ‘lack of regular exercise’ were risk factors of absence. Considering the difficulty of medical treatment in adolescents, education on proper exercise and eating habits is important and may lead to an improvement in premenstrual symptoms and the life qualities of adolescents.

## Abbreviations

CI: confidence interval
OR: odds ratio
PMDD: premenstrual dysphoric disorder
PMS: premenstrual syndrome
PSQ: premenstrual symptoms questionnaire
SD: Standard deviation
SSRI: serotonin selective reuptake inhibitors
